# Passive Smartphone Sensors for Detecting Psychopathology

**DOI:** 10.1001/jamanetworkopen.2025.19047

**Published:** 2025-07-03

**Authors:** Whitney R. Ringwald, Grant King, Colin E. Vize, Aidan G. C. Wright

**Affiliations:** 1 Department of Psychology, University of Minnesota, Minneapolis; 2 Department of Psychology, University of Michigan, Ann Arbor; 3 Department of Psychology, University of Pittsburgh, Pittsburgh, Pennsylvania; 4 Eisenberg Family Depression Center, University of Michigan, Ann Arbor

## Abstract

**Importance:**

Smartphone sensors can continuously and unobtrusively collect clinically relevant behavioral data, allowing for more precise symptom monitoring in clinical and research settings. However, progress in identifying unique behavioral markers of psychopathology from smartphone sensors has been stalled by research on diagnostic categories that are heterogenous and have many nonspecific symptoms.

**Objective:**

To examine which domains of psychopathology are detectable with smartphone sensors and identify passively sensed markers for general impairment (the p-factor) and specific transdiagnostic domains.

**Design, Setting, and Participants:**

This cross-sectional study collected data from the Intensive Longitudinal Investigation of Alternative Diagnostic Dimensions study from January 1 to December 31, 2023, including a baseline survey and 15 days of smartphone monitoring. Participants were recruited from the community via a clinical research registry. A volunteer sample was selected for mental health treatment status.

**Main Outcomes and Measures:**

Transdiagnostic psychopathology dimensions of internalizing, detachment, disinhibition, antagonism, thought disorder, somatoform, and the p-factor; 27 behavior markers derived from a global positioning system, accelerometer, motion, call logs, screen on or off, and battery status.

**Results:**

A total of 557 participants were included in the study (463 [83%] female; mean [SD] age, 30.7 [8.8] years). The coefficient of multiple correlation (*R*) showed that the domain most strongly correlated with sensed behavior was detachment (*R* = 0.42; 95% CI, 0.29-0.54) followed by somatoform (*R* = 0.41; 95% CI, 0.30-0.53), internalizing (R = 0.37), disinhibition (*R* = 0.35; 95% CI, 0.19-0.51), antagonism (*R* = 0.33; 95% CI, 0.6-0.59), and thought disorder (*R* = 0.28; 95% CI, −0.19 to 0.75). Each psychopathology domain was associated with 4 to 10 smartphone sensor variables. Detachment, somatoform, and internalizing had the most behavioral markers. Of the 27 smartphone sensor variables, 14 (52%) had associations with psychopathology domains. After adjusting for shared variance between psychopathology dimensions, all domains except thought disorder retained significant, incremental associations with sensor variables, reflecting unique behavioral signatures (eg, antagonism and number of calls [standardized β = −0.11; 95% CI, −0.20 to −0.02] and disinhibition and battery charge level [standardized β = −0.24; 95% CI, −0.40 to −0.08]). The p-factor was associated with lower mobility (standardized β = −0.22; 95% CI, −0.32 to −0.12), more time at home (standardized β = 0.23; 95% CI, 0.14 to 0.32), later bed time (standardized β = 0.25; 95% CI, 0.11 to 0.38), and less phone charge (standardized β = −0.16; 95% CI, −0.30 to −0.01]). The p-factor was modeled as a latent factor estimated from common variance of the 6 psychopathology domains. All domains loaded moderately to strongly onto the p-factor as expected (standardized loadings: 0.89 for internalizing, 0.76 for somatoform, 0.70 for disinhibition, 0.62 for thought disorder, 0.51 for detachment, and 0.40 for antagonism).

**Conclusions and Relevance:**

This cross-sectional study shows how tethering transdiagnostic domains to concrete behavioral markers can maximize the potential of mobile sensing to study mechanisms driving psychopathology. Insights from these results, and future research that builds on them, can potentially be translated into symptom monitoring tools that fill the gaps in current practice and may eventually lead to more precise and effective treatment.

## Key Points

**Question:**

What forms of psychopathology relate to behavior assessed by smartphone sensors?

**Findings:**

This cross-sectional study of data continuously collected from smartphone sensors from 557 adults for 15 days found that 6 transdiagnostic psychopathology domains were associated with passively sensed behaviors. Passively-sensed behavior markers of the p-factor were also identified.

**Meaning:**

These findings suggest that major forms of psychopathology are detectable from smartphone sensors, indicating that this technology could potentially be used for symptom monitoring and research on wide-ranging psychiatric problems.

## Teaser

This cross-sectional study aimed to establish the breadth of psychopathology in adults that is detectable by smartphone sensors and specificity of markers to different forms of psychopathology.

## Introduction

Digital phenotyping promises to transform clinical assessment and monitoring. Key information about a patient’s symptoms and daily functioning occurring in the days to weeks between clinical encounters is missed with current monitoring practices. In-person clinical observation provides a narrow snapshot of a patient’s status, and patient-reported outcome measures are lengthy and only administered episodically. Dependence on clinical observation and self-report has limited not only clinicians’ ability to monitor symptoms but also scientific knowledge on day-to-day maintenance mechanisms of psychopathology.^1^

Digital phenotyping using mobile sensing can address these limitations. Smartphone sensors continuously and unobtrusively assess behavioral markers of social engagement, physical activity, sleep, and other areas of clinically relevant functioning.^2,3^ Such markers provide concrete, ecologically valid patient data and reveal new insights about the mechanisms driving psychopathology. Initial evidence supports the potential clinical utility of mobile sensing. Behavior detected by smartphone sensors can accurately predict diagnostic status and symptom severity for conditions, including depression,^4,5,6,7,8,9,10,11^ schizophrenia,^12,13^ bipolar disorder,^14,15^ anxiety,^16,17^ and posttraumatic stress disorder.^18,19,20^

Despite the promise of mobile sensing and proliferation of studies in recent years, as with candidate genes and biomarkers, a clear behavioral signature has not been identified for any specific form of psychopathology.^21,22^ Although individual studies support predicting diagnoses or symptoms, each finds a different set of sensed behaviors that are most predictive of a given condition. This lack of progress is in part due to reliance on *Diagnostic and Statistical Manual of Mental Disorders* (Fifth Edition) (*DSM-5*) diagnoses.^23^ One problem with *DSM-5* diagnoses is that they are highly heterogeneous,^24,25,26^ which may explain the inconsistent and even conflicting behavioral markers found for the same disorder in different studies (eg, depression is associated with more^6^ and less^27^ screen time). Another problem is extensive overlap in symptoms across *DSM-5* diagnoses.^28,29,30^ Because nearly all mobile sensing studies have focused on one disorder, it is unknown whether an identified behavioral marker is unique to that form of psychopathology. Indeed, one of the only consistent findings is that every diagnosis investigated to date has been linked to reduced mobility,^21,22,31^ suggesting it is a nonspecific marker of psychopathology. Without a precise and comprehensive mapping of sensed behavior to psychopathology, the potential for digital psychiatry to enhance research and clinical practice cannot be realized.

Dimensional models of psychopathology offer more accurate targets for digital phenotyping. The quantitative empirical structure of psychopathology shows most mental health problems fall within 6 broad domains: internalizing, detachment, disinhibition, antagonism, thought disorder, and somatoform.^32,33,34^ There is also evidence of an even broader general psychopathology dimension (ie, p-factor), reflecting features that cut across all of these domains.^35,36,37^ Transdiagnostic psychopathology dimensions resolve problems of *DSM-5* diagnoses by minimizing within-domain heterogeneity and directly representing the cross-cutting features. Numerous reviews have established that dimensional models improve the ability to identify biomarkers from genetic,^38,39^ electroencephalographic, and structural and functional magnetic resonance imaging^40,41^ data—these same benefits can extend to identifying behavioral markers from smartphone sensors. Namely, organizing behavioral markers into domains that separate shared from specific expressions of psychopathology could address foundational questions of whether sensors only detect general mental health indicators (ie, p-factor) or whether they capture behaviors that differentiate forms of psychopathology (ie, domains) and, if so, the type of behavior.

The current study aims to bring clarity to digital psychiatry by mapping sensed behavioral markers to the 6 major domains of psychopathology and the p-factor. Moreover, because failure to find robust behavioral markers has been compounded by reliance on very small samples (median sample size of 59^21^), we studied one of the largest clinical mobile sensing samples to date. We thereby aimed to establish the breadth of psychopathology that is detectable by smartphone sensors and specificity of markers to different forms of psychopathology.

## Methods

### Participants and Procedures

Sample characteristics and descriptive statistics are given in Table 1. Supplementary materials and data as well as code needed to reproduce our analyses are on the Open Science Framework.^42^ Data came from the Intensive Longitudinal Investigation of Alternative Diagnostic Dimensions study (ILIADD). Study procedures for ILIADD were approved by the University of Pittsburgh institutional review board. Data collection occurred from January 1 to December 31, 2023, in Pittsburgh, Pennsylvania, with all procedures conducted remotely (ie, virtually and by telephone). Participants were recruited through a research recruitment registry supported by the National Institutes of Health’s Clinical and Translational Science Institute. To be eligible, participants had to be between 18 and 50 years of age and own a study-compatible Android or iPhone smartphone. Additionally, participants were selected based on mental health treatment history. Only participants who reported recent treatment were initially eligible to participate in the study, then eligibility criteria were broadened to allow participation regardless of mental health treatment history, ensuring greater variability in symptom presentation (see Table 1 for breakdown of treatment status). Race was self-reported for purposes of characterizing sample demographics. The ILIADD study protocol involved completing a baseline self-report questionnaire battery and optional 15-day ambulatory assessment with smartphone sensor data collection. Race categories included African American or Black, Asian, multiracial, White, and other (Alaska Native or American Indian, Hispanic, Mestiza, Middle Eastern, and Southeast Asian). Written informed consent was obtained before participation. Results reporting adhered to the Strengthening the Reporting of Observational Studies in Epidemiology (STROBE) reporting guideline for cross-sectional studies.

**Table 1.** Characteristics of the Study Participants

Characteristic	No. (%) of participants (N = 557)
Age, mean (SD), y	30.7 (8.8)
Sex	
Female	463 (83)
Male	93 (17)
Intersex	1 (<1)
Race^a^	
African American or Black	29 (5)
Asian	33 (6)
Multiracial	31 (6)
White	456 (82)
Other^b^	8 (1)
Mental health treatment status	
Never in treatment	105 (19)
Currently in treatment	290 (52)
Past-year treatment	56 (10)
Treatment ≥2 years ago	106 (19)
Psychopathology score, mean (SD)^c^	
Internalizing	2.0 (0.7)
Detachment	1.9 (0.7)
Thought disorder	1.2 (0.4)
Disinhibition	1.8 (0.6)
Antagonism	1.4 (0.6)
Somatoform	1.8 (0.6)
Average daily behavior from smartphone sensors, mean (SD)	
GPS	
No. of GPS captures	391.9 (359.5)
No. of travel events	31.5 (28.9)
Total distance traveled, miles	43.4 (60.5)
Maximum distance from home, miles	49.0 (139.1)
Variation in locations visited (log scale)	−7.9 (2.9)
Variation in locations with extended stay (log scale)	−7.3 (1.9)
Time at home, h	14.8 (4.7)
Time spent traveling, h	1.4 (0.9)
Activity (combination of sensors)	
Time driving, h	1.3 (0.9)
Time stationary, h	9.7 (2.6)
Time walking, h	1.2 (0.7)
Time cycling, h	0.1 (0.1)
Time running, h	0.1 (0.1)
Screen on/off	
No. of screen unlocks	76.5 (48.2)
Total time with screen on, h	8.8 (4.3)
Minimum screen on session, h	1.3 (2.8)
Maximum screen on session, h	4.5 (4.03)
Telephone log	
No. of incoming calls	1.5 (1.2)
No. of outgoing calls	2.1 (1.7)
Length of incoming calls, min	10.2 (15.4)
Length of outgoing calls, min	14.8 (27.2)
Battery status	
Time spent charging phone, min	6.0 (2.8)
Minimum battery charge level, %	28.0 (15.0)
Maximum battery charge level, %	91.0 (11.0)
Accelerometer	
Sleep duration, h	7.6 (3.5)
Bedtime hours past midnight	22.5 (1.08)
Wake time hours past midnight	7.5 (1.13)

Abbreviation: GPS, global positioning system.

^a^ Race was self-reported at baseline.

^b^ Identities provided by participants who indicated “other” race included Alaska Native or American Indian, Hispanic, Mestiza, Middle Eastern, and Southeast Asian.

^c^ Psychopathology was measured by the Hierarchical Taxonomy of Psychopathology Patient Reported Outcomes scale.

### Measures

Dimensional psychopathology was assessed at baseline using the development version^1^ of the Hierarchical Taxonomy of Psychopathology Patient Reported Outcomes (HiTOP-PRO) inventory.^43^ The HiTOP-PRO has 405 items assessing past month symptoms and behaviors. Participants rated how much each item applied to them on a scale of 1 (not at all) to 4 (a lot). For the current study, we calculated scores for internalizing, detachment, disinhibition, antagonism, thought disorder, and somatoform. Domain scale items were selected empirically using factor analysis, with a total of 5 to 9 items identified as strong markers per domain. Internal consistency of the scales was adequate (McDonald ω = 0.83-0.89).

Sensor data were collected from participants’ personal smartphones using the Effortless Assessment Research System application.^44^ Data were collected from a global positioning system (GPS), accelerometer, motion, call logs, screen time, and battery status. Longitude and latitude from a GPS were sampled every 10 to 30 seconds, and velocity change from an accelerometer was sampled every second. Motion was inferred from a combination of sensors. Remaining sensors were sampled when a status change was detected. Raw data were then aggregated into 27 variables summarizing daily behavior. A full list of sensor variables is given in Table 1.

Of note, activity (motion sensors) and sleep (accelerometer) variables are indirect measures of behavior and should be interpreted cautiously. Activities (eg, walking) are classified by each operating system’s proprietary algorithm, inferred from a combination of sensors. Accuracy data on these algorithms are not publicly available, but activity classification accuracies reported in the literature are typically above 95%.^45^ Likewise, sleep duration inferred from telephone accelerometer achieves high accuracy^46^; however, it is less accurate than data from wearable technology. To account for differences between Android and iOS classification algorithms, we included operating system as a covariate in models for activity and sleep (n = 119 Android users and 438 iOS users).

### Statistical Analysis

We used multilevel structural equation models (MSEMs)^47^ for our analyses. Analyses were conducted in Mplus, version 8.11 (Muthén & Muthén).^48^ MSEM accommodates the nested structure of the data (ie, days within participants) and allowed us to model individual differences in sensed behavior with a latent intercept. Latent intercepts minimize measurement error and therefore provide a more accurate representation of a person’s typical patterns of behavior compared with calculating an observed person mean.^49^ The statistical tests of interest were regression paths estimating sensed behavior from psychopathology. We considered 2-sided *P* < .05 to indicate statistical significance.

On average, data for a given sensor were missing for participants ranging from 3% (GPS) to 30% (battery status) of their days in the study (all missing data rates reported in eTable 1 in Supplement 1). There are likely unsystematic and systematic reasons for this missing sensor data. Unsystematic reasons (ie, data missing completely at random) include participant’s unintentionally closing the application, technical problems with the application, or the sensors themselves. Systematic reasons (ie, data missing at random) include individual differences, such as disengagement and irresponsibility, that could make some participants more likely to turn off their telephones, let their battery drain, or ignore software or hardware problems. Additionally, call and battery status data were systematically missing for a few of the participants with Android phones (21% of the sample) because these data are unavailable for Android. Thus, because sensor data were assumed to be missing at random or completely at random, missing data were accommodated using full information maximum likelihood techniques in the MSEMs.

## Results

Of the 665 total participants in ILIADD, all analyses in the current study included only those 557 participants (463 [83%] female, 93 [17%] male, and 1 [<1%] intersex; 29 [5%] African American or Black, 33 [6%] Asian, 31 [6%] multiracial, 456 [81%] White, and 8 [1%] other; mean [SD] age, 30.7 [8.8] years) who selected into the ambulatory assessment portion and had sensor data. Compared with participants who only completed the baseline assessment, these participants who opted to complete the ambulatory assessment had slightly lower scores in some psychopathology domains (ie, somatoform, antagonism, and psychoticism) but were no different demographically (Table 1).

Overall strength of association between psychopathology and smartphone sensors was indexed by the coefficient of multiple correlation (*R*) between each domain and the 27 sensor variables. The domain most strongly associated with sensed behaviors was detachment (*R* = 0.42; 95% CI, 0.29-0.54) followed by somatoform (*R* = 0.41; 95% CI, 0.30-0.53), internalizing (*R* = 0.37; 95% CI, 0.25-0.50), disinhibition (*R* = 0.35; 95% CI, 0.19-0.51), antagonism (*R* = 0.33; 95% CI, 0.6-0.59), and thought disorder (*R* = 0.28; 95% CI, −0.19 to 0.75).

**Figure 1.**
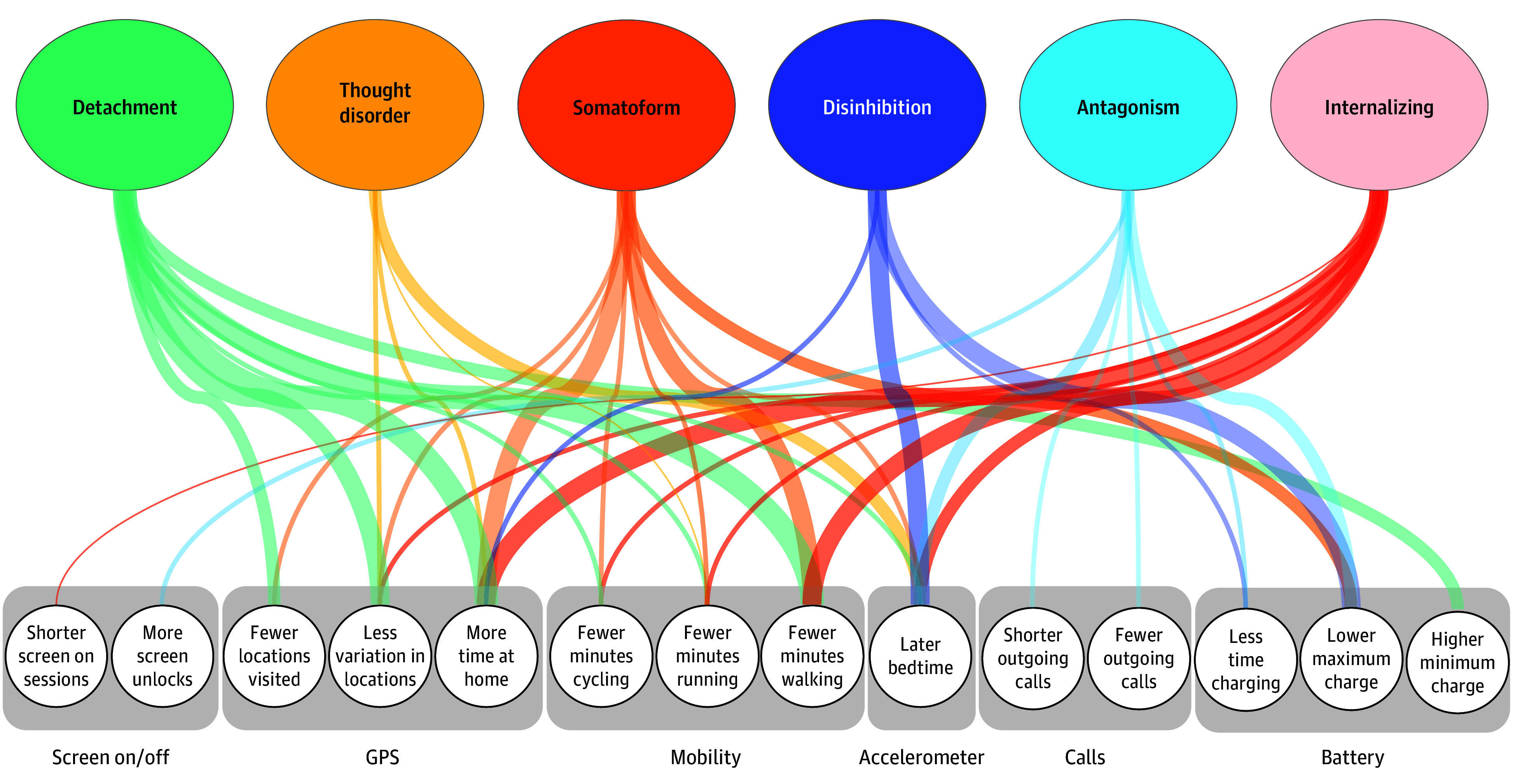
Bivariate Associations Between Psychopathology Domains and Passively Sensed Behavior Markers Width of lines connecting domains to sensors corresponds to strength of associations. GPS indicates global positioning service.

We then identified all behavioral markers of internalizing, detachment, disinhibition, antagonism, thought disorder, and somatoform. In these MSEMs, we estimated bivariate associations by regressing each sensed behavior intercept on a domain. We considered a sensor variable to be a marker for a domain if the association was statistically significant. Table 2 gives the results for the significant bivariate associations, and full results are reported in eTable 2 in Supplement 1. Figure 1 shows these results graphically. Each psychopathology domain was associated with 4 to 10 smartphone sensor variables. Detachment, somatoform, and internalizing had the most behavioral markers. Of the 27 smartphone sensor variables, 14 (52%) had associations with psychopathology domains. Many sensor variables were behavioral markers for multiple domains, justifying the need to separate nonspecific markers (ie, p-factor) from markers unique to a domain.

**Table 2.** Statistically Significant Bivariate and Unique Associations Between Psychopathology and Average Daily Behavior Detected by Smartphone Sensors

Behavioral marker	Bivariate models		Multivariable models (unique associations)	
	Standardized β (95% CI)	*P* value^a^	Standardized β (95% CI)	*P* value^a^
Antagonism				
Bedtime	0.17 (0.04 to 0.29)	.005	0.08 (−0.06 to 0.22)	.27
Maximum battery charge level	−0.15 (−0.28 to −0.01)	.03	−0.09 (−0.23 to 0.05)	.19
No. of outgoing calls	−0.11 (−0.20 to −0.04)	.003	−0.11 (−0.20 to −0.02)	.001
Time spent charging phone	−0.10 (−0.18 to −0.03)	.04	−0.09 (−0.20 to 0.02)	.12
Length of outgoing calls	−0.10 (−0.20 to −0.01)	.008	−0.15 (−0.26 to −0.04)	.002
No. of screen unlocks	0.10 (0.01 to 0.19)	.03	0.16 (0.05 to 0.26)	.003
Detachment				
Time walking	−0.28 (−0.36 to −0.20)	<.001	−0.23 (−0.32 to −0.15)	<.001
Time at home	0.25 (0.15 to 0.34)	<.001	0.18 (0.07 to 0.29)	.001
Variation in locations visited	−0.22 (−0.33 to −0.10)	<.001	−0.19 (−0.32 to −0.06)	.003
No. of travel events	−0.16 (−0.24 to −0.06)	.001	−0.15 (−0.25 to −0.05)	.004
Time running	−0.15 (−0.20 to −0.09)	.02	−0.11 (−0.20 to −0.03)	.01
Minimum battery charge level	0.14 (0.02 to 0.24)	<.001	0.15 (0.03 to 0.27)	.02
Time cycling	−0.13 (−0.24 to −0.03)	.02	−0.07 (−0.20 to 0.06)	.34
Bedtime	0.11 (−0.02 to 0.24)	.03	0.02 (−0.12 to 0.16)	.36
Total distance traveled^a^	−0.08 (−0.19 to 0.01)	.09	−0.15 (−0.25 to −0.05)	.02
Maximum distance from home^a^	−0.08 (−0.17 to 0.01)	.07	−0.08 (−0.22 to −0.02)	.02
Disinhibition				
Maximum battery charge level	−0.21 (−0.34 to −0.08)	.001	−0.24 (−0.40 to −0.08)	.002
Bedtime	0.20 (0.07 to 0.32)	<.001	0.08 (−0.08 to 0.24)	.05
Time at home	0.13 (0.05 to 0.22)	.002	0.02 (−0.10 to 0.14)	.77
Time spent charging phone	−0.12 (−0.22 to −0.02)	.02	−0.13 (−0.26 to 0.00)	.05
Internalizing				
Time walking	−0.20 (−0.29 to −0.11)	<.001	−0.11 (−0.26 to 0.04)	.18
Time at home	0.20 (0.11 to 0.28)	<.001	0.04 (−0.12 to 0.19)	.65
Bedtime	0.21 (0.08 to 0.33)	.004	0.08 (−0.11 to 0.28)	.85
Time cycling	−0.16 (−0.27 to −0.04)	.01	−0.08 (−0.34 to 0.18)	.53
Variation in locations visited	−0.13 (−0.23 to −0.03)	.01	0.00 (−0.19 to 0.19)	.99
Time running	−0.11 (−0.19 to −0.02)	.03	−0.01 (−0.15 to 0.13)	.86
Total time screen on	−0.09 (−0.17 to 0.00)	.04	−0.15 (−0.27 to −0.03)	.01
Maximum screen on session^a^	−0.04 (−0.14 to 0.07)	.50	−0.15 (−0.30 to −0.01)	.03
No. of screen unlocks^a^	−0.01 (−0.11 to 0.09)	.86	0.16 (0.01 to 0.30)	.03
Thought disorder				
Bedtime	0.18 (0.05 to 0.30)	<.001	0.05 (−0.10 to 0.20)	.06
Time at home	0.11 (0.02 to 0.20)	.02	−0.02 (−0.12 to 0.08)	.72
Variation in locations visited	−0.11 (−0.22 to −0.01)	.03	−0.06 (−0.18 to 0.07)	.36
Time running	−0.10 (−0.15 to −0.05)	<.001	−0.07 (−0.14 to 0.01)	.10
Somatoform				
Time walking	−0.21 (−0.29 to −0.13)	<.001	−0.13 (−0.24 to −0.03)	.006
Time at home	0.21 (0.12 to 0.30)	<.001	0.13 (−0.01 to 0.26)	.06
Time cycling	−0.15 (−0.25 to −0.04)	.03	−0.10 (−0.24 to 0.05)	.26
Maximum battery charge level	−0.14 (−0.27 to −0.02)	.007	−0.15 (−0.31 to 0.03)	.09
Variation in locations visited	−0.13 (−0.24 to −0.02)	.02	−0.05 (−0.21 to .010)	.51
Bedtime	0.17 (0.04 to 0.30)	.01	0.01 (−0.15 to 0.18)	.80
No. of travel events	−0.10 (−0.19 to −0.01)	.03	−0.06 (−0.19 to 0.07)	.41
Time running	−0.11 (−0.18 to −0.04)	.005	−0.05 (−0.15 to 0.05)	.43
No. of screen unlocks	−0.06 (−0.16 to 0.03)	.20	−0.13 (−0.26 to 0.00)	.04
p*-*Factor^b^				
Time walking	−0.22 (−0.32 to −0.12)	<.001	NA	NA
Time at home	0.23 (0.14 to 0.32)	<.001	NA	NA
Bedtime	0.25 (0.11 to 0.38)	<.001	NA	NA
Time cycling	−0.17 (−0.28 to −0.06)	.004	NA	NA
Variation in locations visited	−0.16 (−0.27 to −0.05)	.004	NA	NA
Maximum battery charge level	−0.16 (−0.30 to −0.01)	.03	NA	NA
Time running	−0.12 (−0.20 to −0.04)	.004	NA	NA

Abbreviation: NA, not applicable.

^a^ Statistical significance was defined as a 2-sided *P* < .05.

^b^ p-Factor is a latent variable estimated from shared variance of 6 psychopathology domains.

**Figure 2.**
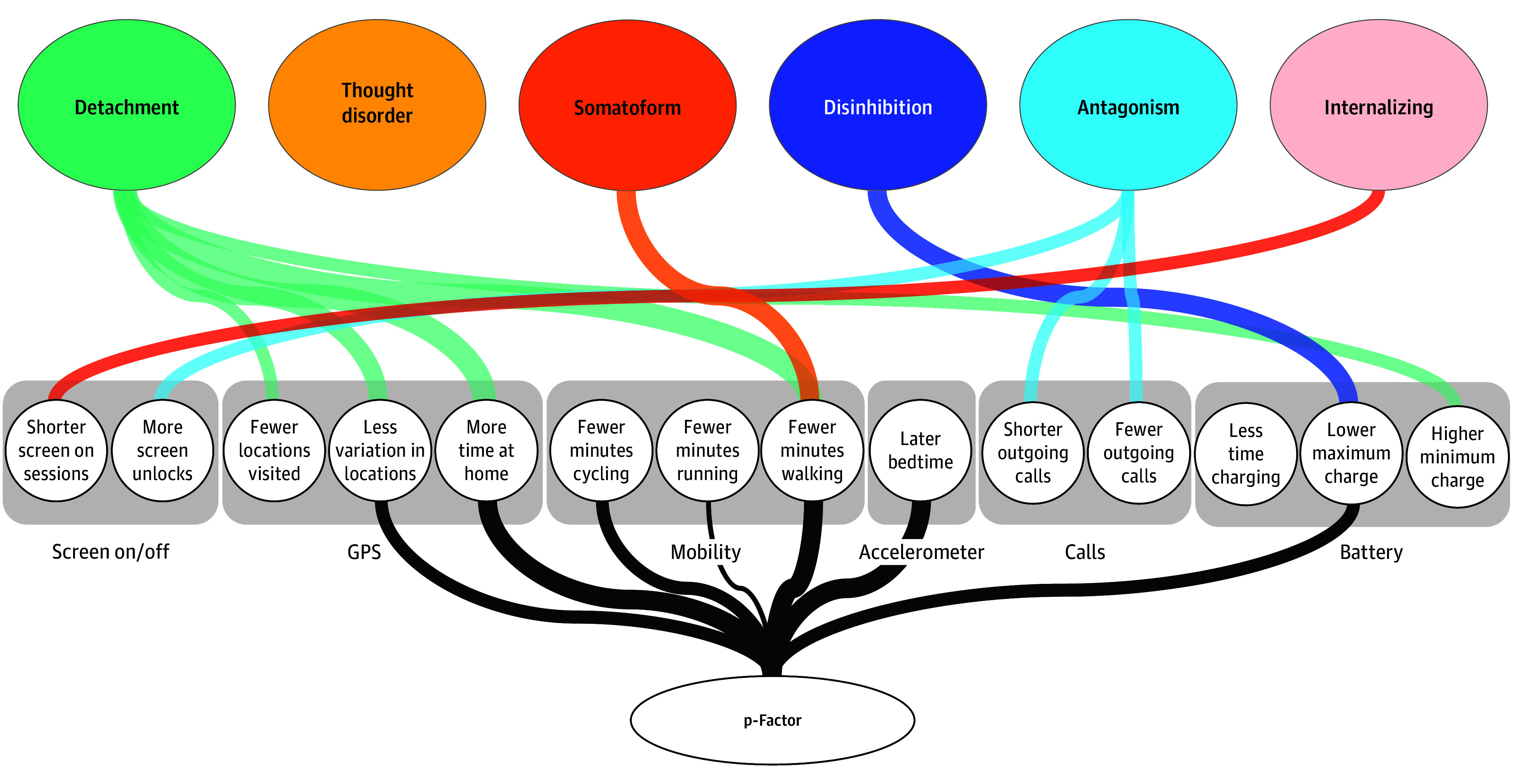
Domain-Specific and p-Factor Associations With Passively Sensed Behavior Markers Width of lines connecting domains to sensors corresponds to strength of associations. Domain-specific associations are incremental associations over and above shared variance among psychopathology domains. GPS indicates global positioning service.

Next, we identified behavioral markers of the p-factor. The p-factor was modeled as a latent factor estimated from common variance of the 6 psychopathology domains. All domains loaded moderately to strongly onto the p-factor as expected (standardized loadings: 0.89 for internalizing, 0.76 for somatoform, 0.70 for disinhibition, 0.62 for thought disorder, 0.51 for detachment, and 0.40 for antagonism). MSEM results in Table 2 and Figure 2 showed that the p-factor was associated with lower mobility (standardized β = −0.22; 95% CI, −0.32 to −0.12), more time at home (standardized β = 0.23; 95% CI, 0.14 to 0.32), later bed time (standardized β = .25; 95% CI, 0.11 to 0.38), and less phone charge (standardized β = −0.16; 95% CI, −0.30 to −0.01]) (results are also plotted in the eFigure in Supplement 1).

Finally, we identified domain-specific markers by entering the 6 domains as simultaneous estimators of each sensor variable in separate MSEMs. Results are given in Table 2 and Figure 2. After adjustment for the shared variance, many associations found in the bivariate models became nonsignificant, indicating that they are nonspecific markers rather than being unique to the domain. Despite this attenuation of associations, all domains except thought disorder had significant incremental associations with some form of passively sensed behavior. Results were generally in line with conceptual expectations; for example, antagonism was incrementally associated with making fewer (standardized β = −0.11; 95% CI, −0.20 to −0.02) and shorter (standardized β = −0.15; 95% CI, −0.26 to −0.04) telephone calls, detachment with lower physical mobility (eg, less time walking: standardized β = −0.23; 95% CI, −0.32 to −0.15]), and disinhibition with maintaining a lower cell phone battery charge (standardized β = −0.24; 95% CI, −0.40 to −0.08).

## Discussion

Reliance on heterogenous and overlapping *DSM-5* diagnoses has stymied advances in digital psychiatry. However, we identified behavioral markers for transdiagnostic domains encompassing most major forms of psychopathology using smartphone sensor data. Furthermore, we found markers of the p-factor and unique behavioral signatures for several domains. These results overcome limitations in the broader literature and help establish the breadth and specificity of psychopathology that can be monitored with mobile sensing and add novel insights into the day-to-day mechanisms driving dysfunction.

Behavioral markers of general psychopathology provide new data to bear on the major outstanding question, “What is the p-factor?”^35,50,51^ To date,^52^ a missing part of the picture has been how the p-factor might manifest in patient behavior. We show that the p-factor had concrete behavioral manifestations: people with higher p-factors in general psychopathology were less physically active, stayed at home more, went to bed later, and charged their telephone less.

The p-factor results clarify that many behaviors identified as markers of specific disorders in prior work are in fact nonspecific markers of psychopathology. Such clarity could only be gained within a transdiagnostic framework, and these findings can inform research and theory on cross-cutting risk factors. For example, later sleep time may be an indicator of evening chronotype, which has been linked to wide-ranging mental health problems.^53^ Likewise, low physical mobility is a measure of sedentariness, which is meta-analytically associated with multiple disorders.^54,55,56^ Other behavioral markers may be indicators of mechanisms hypothesized to explain the p-factor but have been difficult to test empirically. The tendency to stay at home more is consistent with the hypothesis that the p-factor reflects poor emotional constraint, often manifesting in a blunted incentive-approach system and behavioral passivity.^50^ Another hypothesis is that the p-factor reflects impaired cognitive functioning.^35^ Although speculative, the tendency to maintain a lower telephone battery charge may be an indicator of poor planning and broader executive functioning deficits. These possible mappings of behavior markers to mechanisms make hypotheses about the p-factor more tractable and can readily be investigated in future work.

We also identified face valid markers specific to psychopathology domains. These findings both aid interpretation of sensor variables and provide insights into potential maintenance mechanisms. We offer speculative inferences of our findings for illustration but impress that much more work is needed to support these hypotheses. Antagonism incrementally estimated call behavior, which is the most directly social marker available. Results showed that people higher in antagonism tended to make fewer telephone calls—not necessarily receive fewer calls—suggesting that this domain manifests specifically in lower social initiative. Furthermore, calls made by people with higher antagonism scores tended to be shorter, suggesting differences in how they communicate or who they communicate with, with interactions being potentially more transactional than conversational. Consistent with hallmark features of behavioral or social disengagement, we found that people higher in detachment tended to be less physically mobile and stayed at home more. It was also the only domain related to higher minimum battery charge, which could indicate more reliance on relatively passive, telephone-based activities rather than activities outside the home. Alternatively, higher battery charge could reflect low telephone use and disengagement from even electronic activities or social communication. Collecting data on application use in future research would be one approach to disambiguate the meaning of this behavior.

The remaining domains had relatively few unique behavior markers, but those we found are informative. We found that people with higher internalizing scores tended to engage with their telephone in shorter and more frequent bouts (the association with frequency is highly tentative because it was only significant after adjusting for general psychopathology). This finding could indicate anxiety-driven notification checking or brief text communications—possibilities that could be investigated in studies that collect application and text data. The only unique marker of higher disinhibition is lower telephone charge, reinforcing the possibility that this behavior could reflect poor planning and executive functioning difficulties. There is less precedent for the somatoform results. Despite being common and impairing,^57,58^ this domain is understudied.^59^ Our finding that people with higher somatoform scores tended to walk less can be a starting point to form hypotheses and study this pathology if replicated in future research. For example, perhaps (perceived) health problems act as a barrier to physical activity. Overall, these behavioral markers of wide-ranging psychopathology can generate hypotheses and open new lines of inquiry.

The absence of specific behavioral markers for thought disorder is revealing. Previous mobile sensing research has linked schizophrenia to low mobility.^12,13,60,61^ We replicated those findings in bivariate models, but our multivariable models clarified that indexes of low mobility are nonspecific markers of psychopathology. A possible reason that there are no unique markers of thought disorder is that the defining features are cognitive and perceptual, which manifest internally rather than being expressed outwardly in a consistent, detectable manner. Passive sensing technologies exist that are able to capture internal states, for example, detecting language from text and audio, and may be better for assessing this domain.^62,63^

### Limitations

Our study had limitations. First, we examined a relatively limited set of behavioral markers considering the virtually infinite number of variables that can be derived from raw sensor data and considering that there are sensors from which we were unable to collect data (eg, application use). Our success in finding markers of psychopathology despite this limitation is encouraging and suggests that our results represent a lower bound of the ability to identify behavioral markers with mobile sensing. Research incorporating more sensors and work developing variables that optimize estimation or explainability will likely lead to discovery of even more comprehensive behavioral markers. Second, our inferences about the mechanistic role of behavioral markers were limited due to the observational study design and short study duration. However, these markers provide a strong starting point to inform hypotheses about mechanisms to be tested in future work. Third, sample characteristics may limit generalizability of our results. Namely, the sample was mostly female. Additionally, although our sample was enriched for people who reported recent mental health treatment, our findings may not generalize to more severe clinical populations or some forms of underrepresented psychopathology (eg, thought disorder). Research in samples balanced on sex and including participants with higher acuity is needed to identify behavioral markers spanning the full range of severity in dimensional psychopathology. Fourth, the extent to which behavioral markers are influenced by psychopathology-irrelevant factors, such whether participants keep their telephone in a pocket vs a purse, is unknown. Lack of control over participant behavior is an inherent trade-off in naturalistic passive sensing studies, and focused research is needed to determine how much such behaviors affect results.

## Conclusions

In this cross-sectional study, we identified behavioral markers for domains encompassing most major forms of psychopathology using smartphone sensor data. In addition to establishing the breadth of psychopathology that was detectable, findings show smartphone sensors assessed markers that distinguish domains of dysfunction. Results showing many behavioral markers reflected that nonspecific psychopathology reinforces the need for dimensional, transdiagnostic models to maximize the potential of mobile sensing technology. These results suggest that the findings from this study may advance research on day-to-day maintenance mechanisms of psychopathology and inform development of symptom monitoring tools.

## Notes

Supplement 1. **eTable 1.** Rates of Missingness Per Sensor Variable **eTable 2.** Full Multilevel Structural Equation Model Results **eFigure.** Association Between the p-Factor and Average Bedtime

Supplement 2.
Data Sharing Statement
