# Supplementary material for: Passive Smartphone Sensors for Detecting Psychopathology
Source: JAMA Netw Open. 2025 Jul 3;8(7):e2519047. doi: 10.1001/jamanetworkopen.2025.19047 (PMC12232220; doi:10.1001/jamanetworkopen.2025.19047)
Supplement: Supplement 1. — eTable 1. Rates of Missingness Per Sensor Variable eTable 2. Full Multilevel Structural Equation Model Results eFigure. Association Between the p-Factor and Average Bedtime [file jamanetwopen-e2519047-s001.pdf]

Supplemental Online Content

Ringwald WR, King G, Vize CE, Wright AGC. Passive smartphone sensors for detecting psychopathology. *JAMA Netw Open*. 2025;8(7):e2519047.
doi:10.1001/jamanetworkopen.2025.19047

eTable 1. Rates of Missingness Per Sensor Variable

eTable 2. Full Multilevel Structural Equation Model Results

eFigure. Association Between the p-Factor and Average Bedtime

This supplemental material has been provided by the authors to give readers additional information about their work.

© 2025 Ringwald WR et al. *JAMA Network Open.*

eTable 1. Rates of Missingness Per Sensor Variable

Sensor variable	Average % days missing per person
**Accelerometer**	
Bed time	16
Wake time	16
Sleep duration	16
**Battery status**	
Time spent charging	26
Minimum battery charge	26
Maximum battery charge	26
**Phone log**	
Number of incoming calls	30
Number of outgoing calls	30
Length of incoming calls (minutes)	30
Length of outgoing calls (minutes)	30
**GPS**	
Number of GPS captures	2
Number of travel events	5
Total distance travelled (miles)	2
Maximum distance from home (miles)	2
Variation in locations visited (log scale)	13
Variation in locations with extended stay (log scale)	13
Time at home (hours)	3
Time spent travelling (hours)	7
**Activity**	
Time driving (hours)	15
Time stationary (hours)	15
Time walking (hours)	15
Time cycling (hours)	15
Time running (hours)	15
**Screen on/off**	
Number of screen unlocks	24
Total time with screen on (hours)	24
Minimum screen on session (hours)	24
Maximum screen on session (hours)	24

Note. N = 557; Battery and phone calls were only available for participants with iOS (*N* = 438).

© 2025 Ringwald WR et al. *JAMA Network Open.*

eTable 2. Full Multilevel Structural Equation Model Results

Sensor	Bivariate beta	*p*-value	Multivariable beta	*p*-value
**Antagonism**				
Number of outgoing calls	-.132	.003	-.153	.001
Bed time	-.152	.005	-.064	.265
Length of outgoing calls	-.102	.008	-.172	.002
Number of screen unlocks	.102	.027	.15	.003
Maximum battery charge level	-.144	.034	-.09	.19
Time spent charging phone	-.103	.039	-.085	.123
Number of GPS captures	-.079	.08	-.068	.219
Number of incoming calls	.096	.166	.105	.123
Total time with screen on	-.055	.213	-.042	.401
Time cycling	-.053	.244	-.007	.887
Time at home	.048	.311	-.035	.493
Total time with screen on	.046	.361	.021	.69
Variation in locations visited	-.042	.381	-.066	.224
Sleep duration	-.045	.396	-.025	.67
Time driving	-.037	.448	-.091	.181
Number of GPS captures	-.033	.516	-.032	.561
Wake time	.035	.53	.044	.474
Time walking	-.024	.62	.033	.511
Number of incoming calls	-.015	.764	.016	.76
Minimum battery charge level	.012	.82	.012	.84
Time stationary	-.011	.86	-.023	.708
Number of travel events	-.008	.869	.041	.454
Time running	-.007	.869	.041	.422
Maximum screen on session	.005	.92	-.012	.825
Maximum distance from home	-.001	.973	-.005	.904
Variation locations, extended stay	-.001	.988	.06	.244
Total distance travelled	-.001	.989	-.034	.566
**Detachment**				
Time at home	.245	.000	.186	.001
Variation in locations with extended stay	-.218	.000	-.192	.003
Time running	-.138	.000	-.109	.02
Time walking	-.308	.000	-.25	.000

© 2025 Ringwald WR et al. *JAMA Network Open.*

Number of GPS captures	-.155	.001	-.148	.004
Time cycling	-.124	.019	-.065	.343
Minimum battery charge level	.14	.021	.162	.014
Bed time	-.124	.027	-.056	.362
Maximum distance from home	-.064	.074	-.081	.024
Wake time	-.089	.085	-.069	.295
Number of screen unlocks	-.082	.09	-.07	.188
Total distance travelled	-.08	.093	-.119	.021
Number of travel events	-.069	.159	-.048	.377
Number of GPS captures	-.049	.293	-.065	.218
Time spent charging phone	-.053	.319	-.033	.584
Total time with screen on	.049	.376	.105	.077
Total time with screen on	.048	.387	.048	.432
Time driving	.043	.446	.014	.82
Sleep duration	.032	.515	.079	.168
Maximum battery charge level	-.04	.555	.013	.858
Maximum screen on session	.032	.563	.05	.425
Time stationary	.034	.588	.041	.533
Number of incoming calls	-.023	.654	-.015	.785
Number of incoming calls	-.032	.684	-.048	.491
Number of outgoing calls	.023	.723	-.009	.89
Variation in locations visited	-.017	.761	-.036	.529
Length of outgoing calls	.022	.819	-.011	.894
**Disinhibition**				
Bed time	-.203	.000	-.133	.045
Maximum battery charge level	-.219	.001	-.247	.002
Time at home	.137	.002	.019	.768
Time spent charging phone	-.125	.021	-.134	.046
Number of screen unlocks	-.1	.046	-.177	.003
Variation in locations with extended stay	-.079	.112	.011	.875
Time walking	-.079	.121	.096	.119
Number of GPS captures	-.069	.136	-.012	.858
Total time with screen on	.076	.141	.101	.114
Time cycling	-.075	.149	.035	.696
Number of travel events	-.064	.164	-.058	.374
Time running	-.052	.265	.021	.731
Time stationary	.067	.288	.106	.155
Maximum screen on session	.045	.349	.097	.098
Minimum battery charge level	-.053	.359	-.128	.077
Total time with screen on	-.032	.393	.034	.486
Wake time	.042	.41	.14	.039

© 2025 Ringwald WR et al. *JAMA Network Open.*

Number of GPS captures	-.036	.44	-.064	.351
Sleep duration	-.038	.441	.01	.872
Length of outgoing calls	.06	.5	.079	.319
Maximum distance from home	-.033	.513	-.043	.698
Number of incoming calls	-.034	.527	-.068	.365
Time driving	.027	.572	.008	.913
Number of incoming calls	.037	.66	.014	.857
Variation in locations visited	-.007	.894	-.006	.932
Total distance travelled	.008	.907	.003	.984
Number of outgoing calls	-.004	.941	-.031	.684
**Internalizing**				
Time at home	.198	.000	.037	.649
Time walking	-.22	.000	-.104	.18
Bed time	-.161	.004	.017	.845
Time cycling	-.145	.013	-.085	.525
Variation in locations with extended stay	-.129	.014	.000	.997
Time running	-.097	.026	-.013	.857
Total time with screen on	-.091	.044	-.155	.013
Number of GPS captures	-.082	.114	.014	.871
Maximum battery charge level	-.086	.168	.15	.085
Wake time	-.07	.179	-.114	.202
Sleep duration	-.067	.186	-.105	.182
Number of outgoing calls	.074	.262	.164	.138
Number of travel events	-.044	.33	.093	.247
Time spent charging phone	-.049	.378	.075	.396
Maximum screen on session	-.037	.501	-.157	.032
Time driving	.04	.514	-.045	.641
Length of outgoing calls	.04	.641	-.014	.865
Number of incoming calls	.026	.669	.192	.061
Number of incoming calls	.028	.742	.038	.673
Maximum distance from home	-.017	.751	-.024	.794
Minimum battery charge level	.018	.771	-.002	.981
Time stationary	.015	.807	.008	.922
Number of screen unlocks	-.009	.858	.16	.025
Number of GPS captures	.007	.882	.087	.325
Variation in locations visited	.006	.913	-.028	.736
Total time with screen on	.005	.92	-.134	.081
Total distance travelled	.006	.928	-.032	.787
**Thought Disorder**				
Time running	-.092	.000	-.062	.102
Bed time	-.193	.000	-.109	.062

© 2025 Ringwald WR et al. *JAMA Network Open.*

Time at home	.113	.021	-.02	.716
Variation in locations with extended stay	-.112	.032	-.059	.359
Time cycling	-.076	.075	.013	.809
Time walking	-.079	.098	.059	.334
Number of travel events	-.062	.202	-.036	.548
Minimum battery charge level	.063	.212	.076	.228
Number of outgoing calls	-.05	.315	-.048	.41
Time driving	.071	.324	.085	.371
Total time with screen on	.053	.344	.034	.612
Length of outgoing calls	.068	.416	.117	.146
Total distance travelled	.045	.422	.058	.38
Wake time	-.037	.449	-.029	.621
Sleep duration	-.039	.463	-.005	.942
Maximum battery charge level	-.052	.488	.086	.304
Number of incoming calls	-.033	.509	-.024	.644
Maximum screen on session	.033	.56	.048	.487
Number of incoming calls	.026	.58	-.025	.72
Time spent charging phone	-.034	.605	.05	.494
Number of GPS captures	-.019	.786	.075	.361
Number of screen unlocks	.011	.8	.025	.607
Total time with screen on	-.009	.835	.054	.28
Number of GPS captures	.01	.86	.037	.607
Variation in locations visited	.008	.898	.015	.847
Time stationary	.002	.969	-.006	.918
Maximum distance from home	.000	.996	.012	.64
**Somatoform**				
Time at home	.209	.000	.126	.061
Time walking	-.236	.000	-.153	.006
Time running	-.096	.005	-.04	.427
Time cycling	-.146	.007	-.083	.259
Bed time	-.129	.014	.017	.799
Variation in locations with extended stay	-.13	.019	-.053	.507
Maximum battery charge level	-.156	.024	-.15	.088
Number of GPS captures	-.097	.029	-.055	.406
Number of travel events	-.091	.041	-.105	.125
Number of incoming calls	-.084	.088	-.174	.017
Total time with screen on	-.068	.147	-.023	.705
Number of screen unlocks	-.067	.194	-.135	.04
Time driving	.071	.204	.084	.36
Time spent charging phone	-.067	.249	-.034	.675

© 2025 Ringwald WR et al. *JAMA Network Open.*

Wake time	-.054	.283	-.017	.808
Variation in locations visited	.045	.39	.099	.134
Total distance travelled	.051	.435	.104	.208
Sleep duration	-.038	.44	.009	.893
Total time with screen on	.043	.45	.049	.505
Maximum distance from home	.031	.571	.096	.07
Time stationary	-.037	.574	-.098	.233
Number of outgoing calls	.025	.628	.007	.925
Length of outgoing calls	.031	.695	.03	.665
Minimum battery charge level	.022	.704	-.011	.897
Number of GPS captures	-.016	.728	-.026	.708
Number of incoming calls	.009	.912	-.035	.675
Maximum screen on session	.002	.977	.031	.662
***p*-factor**				
Time at home	.233	.000		
Time walking	-.244	.000		
Bed time	-.211	.000		
Variation in locations with extended stay	-.158	.004		
Time cycling	-.16	.004		
Time running	-.115	.004		
Maximum battery charge level	-.157	.032		
Number of GPS captures	-.107	.047		
Total time with screen on	-.079	.112		
Number of travel events	-.076	.116		
Time spent charging phone	-.089	.152		
Sleep duration	-.061	.263		
Wake time	-.057	.287		
Time driving	.058	.342		
Total time with screen on	.045	.454		
Number of screen unlocks	-.04	.47		
Number of outgoing calls	.037	.575		
Minimum battery charge level	.028	.658		
Length of outgoing calls	.045	.661		
Number of incoming calls	.034	.727		
Number of incoming calls	-.019	.742		
Maximum distance from home	-.014	.79		
Total distance travelled	.016	.801		
Number of GPS captures	-.012	.824		
Time stationary	.014	.835		
Variation in locations visited	.01	.866		
Maximum screen on session	-.005	.929		

© 2025 Ringwald WR et al. *JAMA Network Open.*

Note. *N* = 557. Multivariable beta is from multivariable regression with all psychopathology domains regressed on a sensor in separate models.

eFigure. Association Between the p-Factor and Average Bedtime

© 2025 Ringwald WR et al. *JAMA Network Open.*

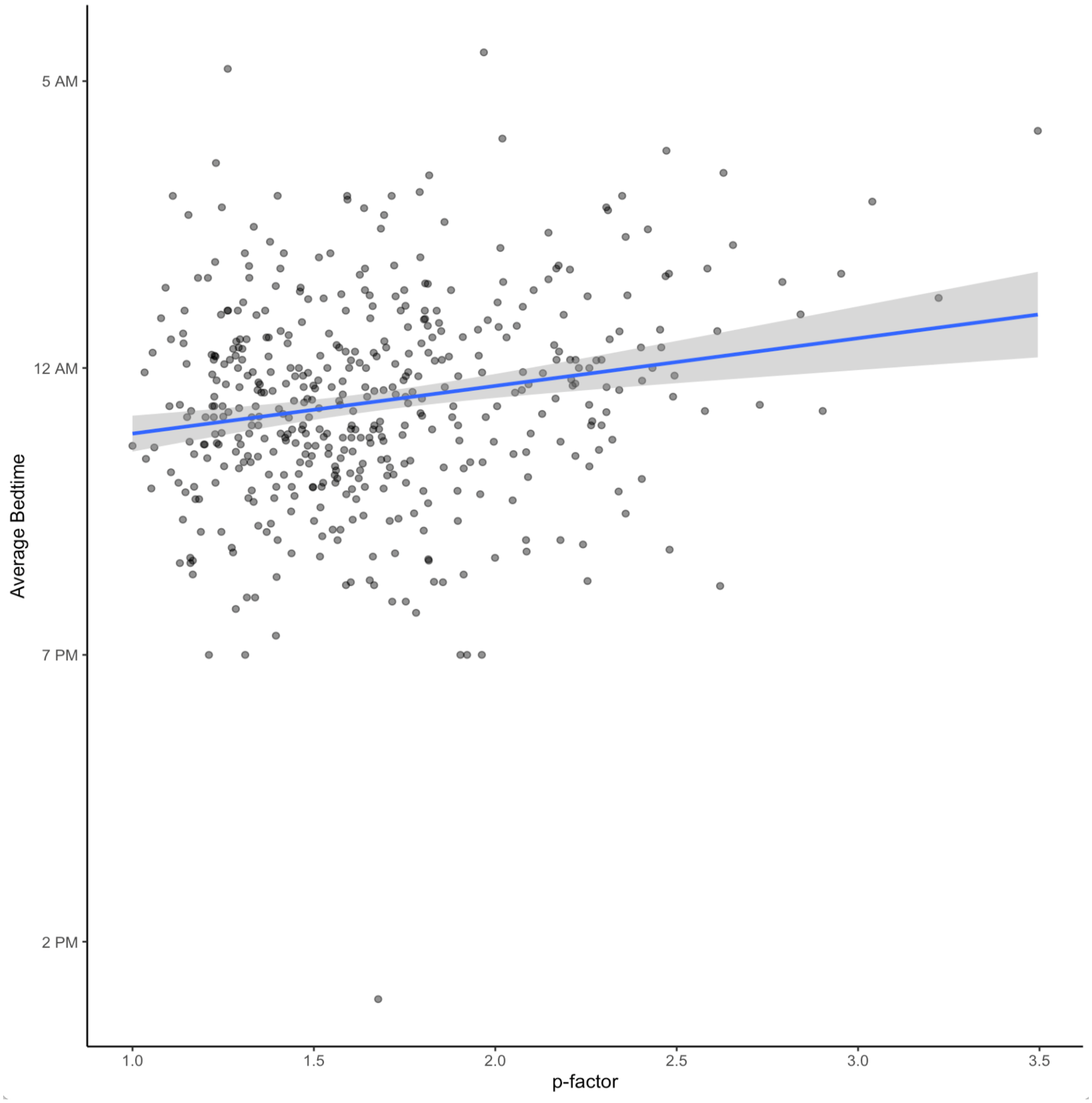

Note. Latent means and *p*-factor from multilevel structural equation models (MSEMs) were used for the main analyses, observed average bedtime and observed *p*-factor were used for the purpose of visualization. *p*-factor was calculated by the mean of all psychopathology scales. Standardized beta from MSEMs = .25 [.11-.38].

© 2025 Ringwald WR et al. *JAMA Network Open.*
