# Supplementary material for: Passive Smartphone Sensors for Detecting Psychopathology
Source: JAMA Netw Open. 2025 Jul 3;8(7):e2519047. doi: 10.1001/jamanetworkopen.2025.19047 (PMC12232220; doi:10.1001/jamanetworkopen.2025.19047)
Supplement: Supplement 2. — Data Sharing Statement [file jamanetwopen-e2519047-s002.pdf]

Data Sharing Statement

Ringwald. Passive Smartphone Sensors for Detecting Psychopathology. *JAMA Netw Open*. Published July 03, 2025. doi:10.1001/jamanetworkopen.2025.19047

Data
Data available: Yes
Data types: Deidentified participant data
How to access data: osf.io/qwpcx/
When available: With publication

Supporting Documents
Document types: Statistical/analytic code
How to access documents: osf.io/qwpcx/
When available: With publication

Additional Information
Who can access the data: Publicly available
Types of analyses: Publicly available
Mechanisms of data availability: Publicly available
